# Detecting depressive and anxiety disorders in distressed patients in primary care; comparative diagnostic accuracy of the Four-Dimensional Symptom Questionnaire (4DSQ) and the Hospital Anxiety and Depression Scale (HADS)

**DOI:** 10.1186/1471-2296-10-58

**Published:** 2009-08-23

**Authors:** Berend Terluin, Evelien PM Brouwers, Harm WJ van Marwijk, Peter FM Verhaak, Henriëtte E van der Horst

**Affiliations:** 1 Department of General Practice, EMGO Institute for Health and Care Research, VU University Medical Centre, Amsterdam, the Netherlands; 2 Academic Centre for Transformation in Care and Welfare (TRANZO), Tilburg University, Tilburg, the Netherlands; 3 Netherlands Institute for Health Services Research (NIVEL), Utrecht, the Netherlands

## Abstract

**Background:**

Depressive and anxiety disorders often go unrecognized in distressed primary care patients, despite the overtly psychosocial nature of their demand for help. This is especially problematic in more severe disorders needing specific treatment (e.g. antidepressant pharmacotherapy or specialized cognitive behavioural therapy). The use of a screening tool to detect (more severe) depressive and anxiety disorders may be useful not to overlook such disorders. We examined the accuracy with which the Four-Dimensional Symptom Questionnaire (4DSQ) and the Hospital Anxiety and Depression Scale (HADS) are able to detect (more severe) depressive and anxiety disorders in distressed patients, and which cut-off points should be used.

**Methods:**

Seventy general practitioners (GPs) included 295 patients on sick leave due to psychological problems. They excluded patients with recognized depressive or anxiety disorders. Patients completed the 4DSQ and HADS. Standardized diagnoses of DSM-IV defined depressive and anxiety disorders were established with the Composite International Diagnostic Interview (CIDI). Receiver Operating Characteristic (ROC) analyses were performed to obtain sensitivity and specificity values for a range of scores, and area under the curve (AUC) values as a measure of diagnostic accuracy.

**Results:**

With respect to the detection of any depressive or anxiety disorder (180 patients, 61%), the 4DSQ and HADS scales yielded comparable results with AUC values between 0.745 and 0.815. Also with respect to the detection of moderate or severe depressive disorder, the 4DSQ and HADS depression scales performed comparably (AUC 0.780 and 0.739, p 0.165). With respect to the detection of panic disorder, agoraphobia and social phobia, the 4DSQ anxiety scale performed significantly better than the HADS anxiety scale (AUC 0.852 versus 0.757, p 0.001). The recommended cut-off points of both HADS scales appeared to be too low while those of the 4DSQ anxiety scale appeared to be too high.

**Conclusion:**

In general practice patients on sick leave because of psychological problems, the 4DSQ and the HADS are equally able to detect depressive and anxiety disorders. However, for the detection of cases severe enough to warrant specific treatment, the 4DSQ may have some advantages over the HADS, specifically for the detection of panic disorder, agoraphobia and social phobia.

## Background

Depressive and anxiety disorders are prevalent in primary care patients, although these disorders are often not recognized as such by the general practitioner (GP) [1-3]. One explanation for non-recognition of psychiatric disorders concerns the time constraints in general practice, and the competing demands put upon the GP [4]. A special situation occurs in patients with an overt psychosocial presentation. These distressed patients present with psychological complaints, such as nervousness or feeling depressed, or psychosocial problems, such as occupational or marital problems, which are readily available for discussion with the GP [5]. Because these complaints and problems often arise in the context of 'life stress', the discussion usually remains exclusively focused on psychosocial problems, not on complaints and symptoms that might indicate a specific psychiatric disorder. In order to diagnose such disorders, GPs need to make a clinical assessment of certain symptoms. However, it is not always easy for the GP to change the subject of the discussion away from the (presumed) causes of the symptoms, towards an assessment of the symptoms themselves. Therefore, whereas GPs are fully aware of the presence of psychological problems, they may fail to establish specific psychiatric diagnoses of depressive and anxiety disorders in many cases where such diagnoses are justified [6]. This is especially problematic in more severe psychiatric disorders since these often require specific treatment. The use of questionnaires can be a useful strategy to detect (more severe) depressive and anxiety disorders in patients with relatively high risks, i.e. in patients presenting with distress in general practice [7].

In this study we examined and compared the diagnostic accuracy of two questionnaires, the Four-Dimensional Symptom Questionnaire (4DSQ) and the Hospital Anxiety and Depression Scale (HADS), with respect to the detection of DSM-IV defined depressive and anxiety disorders in a sample of distressed patients in primary care with and without such disorders. In distressed patients we do not need questionnaires to detect mild depressive or anxiety disorders (let alone depressive or anxiety symptoms) since these patients often need no other interventions than non-specific counselling or problem-solving focused on how to cope with the stress in the patients' lives [8]. On the other hand, it is specifically important to identify patients with more severe disorders who may be in need of specific treatment (e.g. antidepressant pharmacotherapy or specialized cognitive behavioural therapy). Therefore, we did not only look at how well the 4DSQ and the HADS detected any DSM-IV defined depressive and anxiety disorders but we also analyzed, more specifically, how well these questionnaires detected moderate and severe major depressive disorders, panic disorder, agoraphobia and social phobia.

## Methods

### Patients and procedure

We analyzed cross-sectionally baseline data collected for a randomized controlled trial concerning an activating social work intervention for distressed patients in general practice [9]. Patients were included in the study if they were aged 18–60, had a 'nervous breakdown' according to the GP, were employed, and had been on sick leave for no longer than three months. Dutch GPs use the diagnostic label 'nervous breakdown' (or 'overstressed', in Dutch 'overspanning') for a syndrome that is associated with too much stress to the extent that the patient cannot cope anymore [10,11]. This condition is characterized by psychological distress, failure to cope, and social dysfunctioning (i.e. sick leave in employed people) [12]. The GPs were instructed not to include patients with obvious depressive and anxiety disorders (i.e. patients in whom they had clinically diagnosed such disorders). Adequate command of the Dutch language, and no current psychological treatment were additional inclusion criteria. Between August 2001 and July 2003, 70 GPs in the city of Almere, the Netherlands, assessed and referred 370 patients to the study centre. The patients were contacted by telephone, information about the study was given and inclusion criteria were checked. Thirty patients were unwilling to participate, and a further 33 patients did not meet the inclusion criteria. With the remaining patients an appointment was made for a baseline interview in their homes and they were sent written information and baseline questionnaires by post. The home visit took place on average 5.7 days after the initial telephone contact. After the patients had been fully informed about the study, written informed consent was obtained. The participating patients (n = 307) were then interviewed, and subsequently their questionnaires were checked for missing values by the interviewer. Unfortunately, the date at which the questionnaires were completed was not recorded. Therefore, the exact time interval between the questionnaires and the interviews is unknown, but we estimate it to be on average between 2 and 5 days. Because 12 patients failed to complete the questionnaires, the sample for the present study comprised 295 patients. Prior to the start of the study, approval was obtained from the ethical committee of the Netherlands Institute of Mental Health and Addiction.

### Measurements

The interview comprised the mood and anxiety disorder sections of the Composite International Diagnostic Interview (CIDI), a standardized diagnostic interview, developed to be applied by trained lay interviewers, resulting in psychiatric diagnoses according to DSM-IV and ICD-10 criteria [13]. The specific phobia section was omitted because these problems are (if not accompanied by other mental disorders) associated with relatively little disability and impairment [14,15] and, therefore, isolated (non-comorbid) specific phobia appears to be of relatively little importance in patients with a 'nervous breakdown'. The obsessive-compulsive disorder section was omitted because of the low prevalence of this disorder [14,16]. Accordingly, the present study used the following current DSM-IV [17] diagnoses: major depressive disorder, dysthymia, bipolar disorder, generalized anxiety disorder, panic disorder with and without agoraphobia, agoraphobia without panic disorder, and social phobia. The CIDI was administered by five interviewers who received a training course at the Dutch WHO-CIDI Training and Reference Centre at Amsterdam, after which they were certified to deliver the fully structured CIDI interview. They used a computer-assisted version in which the questions were presented according to diagnostic algorithms and responses were entered directly into the computer. None of the interviewers had any particular expertise in psychology or psychiatry. The interviewers remained ignorant of the CIDI-diagnoses as such.

The questionnaires encompassed the Four-Dimensional Symptom Questionnaire (4DSQ) and the Hospital Anxiety and Depression Scale (HADS). The 4DSQ is a 50-item self-rating questionnaire measuring 'distress', 'depression', 'anxiety' and 'somatization' [18]. The 4DSQ assesses psychological and psychosomatic symptoms experienced during the past seven days. The distress scale (16 items, score range 0–32) measures symptoms of general psychological distress, which is conceptualized as the most general, most basic expression of human psychological suffering [18,19]. The depression scale (6 items, score range 0–12) measures severe anhedonia and depressive cognitions (including suicidal ideation), symptoms considered to be characteristic of depressive disorder [20,21]. The anxiety scale (12 items, score range 0–24) measures irrational fears, panic and avoidance, characteristic features of most anxiety disorders [17]. The somatization scale (16 items, score range 0–32) measures a range of 'psychosomatic' symptoms, characteristic of bodily distress and somatoform disorders [22]. For all 4DSQ scales, higher scores represent higher symptom levels. In this study, we examined the 4DSQ distress scale for its ability to detect any depressive or anxiety disorder, and the 4DSQ depression and anxiety scales for their abilities to detect depressive and anxiety disorders. The 4DSQ depression and anxiety scales are supposed to detect depressive and anxiety disorders severe enough to consider specific treatment. For the 4DSQ scales two cut-off points are recommended, dividing the scales into low, moderate and high scores (Table 1). High scores indicate a relatively high probability of caseness, prompting an immediate clinical diagnosis. On the other hand, low scores indicate the probable absence of a clinically relevant disorder. Moderate scores indicate a relatively low probability of caseness, warranting follow-up and reassessment after a few weeks [7].

**Table 1:** Recommended cut-off points for the 4DSQ and HADS depression and anxiety scales*

**Instrument**	**Scale**	**Lower cut-off point**	**Higher cut-off point**
4DSQ	depression	≥3	≥6

	anxiety	≥8	≥13

HADS	depression	≥8	≥11

	anxiety	≥8	≥11

* Scores below the lower cut-off point indicate the probable absence of a disorder, scores between the lower and higher cut-off points indicate the possible existence of a disorder, and scores exceeding the higher cut-off point indicate the probable existence of a disorder.

4DSQ = Four-Dimensional Symptom Questionnaire

HADS = Hospital Anxiety and Depression Scale

The HADS is a 14-item self-rating questionnaire measuring 'depression' and 'anxiety' [23-26]. Like the 4DSQ, the HADS uses seven days as reference period. The depression scale (7 items, score range 0–21) measures mostly anhedonia, a phenomenon considered to be the central characteristic of major depressive disorder [21]. The anxiety scale (7 items, score range 0–21) measures mostly symptoms of generalized anxiety disorder [27]. For both HADS scales higher scores represent higher symptom levels. The depression and anxiety scales are intended to detect depressive and anxiety disorders in general medical settings. The depression scale is specifically intended to select those depressed patients which may be helped by the prescription of an antidepressant drug [21]. Like the 4DSQ, the HADS scales employ two cut-off points, one for the detection of 'possible' and one for the detection of 'probable' depressive or anxiety disorder (Table 1). Because the HADS total score is sometimes recommended as a measure of general psychological distress [28,29], we examined the total score for its ability to detect any depressive or anxiety disorder.

There is a noteworthy difference in item content between the 4DSQ and the HADS. Unlike the 4DSQ, the HADS contains six positively worded items, of which five belong to the depression scale (e.g. 'I can laugh and see the funny side of things'; 'I feel cheerful'). These five depression items are assumed to measure anhedonia (i.e. loss of the ability to enjoy ordinary things in life) if the scores are reversed. In contrast, the 4DSQ depression scale heavily rests on depressive cognitions, including suicidal ideation (e.g. 'did you feel that everything is meaningless?'; 'did you ever think "If only I was dead"?'). At first glance, the 4DSQ depression scale seems to tap more severe depressive symptoms than the HADS depression scale. Regarding anxiety, unlike the HADS anxiety scale, the 4DSQ anxiety scale contains items on phobic fears and avoidance behaviour (e.g. 'were you afraid to travel on busses, trains or trams?'; 'were you afraid of becoming embarrassed when with other people?'; 'did you have to avoid certain places because they frightened you?'). In contrast, the HADS anxiety scale contains a few items of which the equivalents are included in the 4DSQ distress scale (e.g. 'feeling tense', 'worrying', 'feeling restless'). Again, at first glance, the 4DSQ anxiety scale seems to tap more severe symptoms than the HADS anxiety scale.

The interviewers collected the questionnaires after the CIDI-interview and checked the questionnaires for missing values. They did not have the knowledge, means or time to calculate scale scores. The questionnaire scores were later entered into a database by research assistants who were ignorant of the CIDI diagnoses.

### Analysis

First, we calculated Cronbach's α values as a measure of internal consistency and an estimate of reliability, and mean 4DSQ and HADS scores for the various diagnostic categories.

Second, we performed Receiver Operating Characteristic (ROC) analyses and estimated area under the curve (AUC) values as a measure of diagnostic accuracy. The ROC curve is a graphical representation of the sensitivity and 1-specificity values of all possible cut-off values of a continuous diagnostic variable [30]. The AUC value represents the probability that a randomly chosen case has a higher score than a randomly chosen non-case [31]. Differences between AUC values were tested using the method outlines by Hanley and McNeil [32].

Third, we calculated sensitivity, specificity, positive predictive values (PPV) and negative predictive values (NPV) for a range of relevant cut-off points for each scale. All analyses were performed with SPSS 14.0.

## Results

### Description of the sample

The sample consisted of 178 women and 117 men with a mean age of 39.5 years (SD 9.2). Dysthymia was diagnosed only in seven patients of whom six also fulfilled criteria for major depressive disorder. Bipolar disorder was diagnosed only in five patients who all had a 'mild manic episode'. Because of the small numbers, we decided not to include dysthymia and bipolar disorder in the further analyses (but we did not exclude the patients). Most patients with a major depressive disorder had a single episode; only 11% were diagnosed as having a recurrent disorder. Table 2 provides details about the distribution of the CIDI/DSM-IV diagnoses, as well as the internal consistency of the questionnaire scales, and the mean scores of the scales per diagnostic category. Not surprisingly, patients with the diagnosis of severe major depression had the highest scores on the 4DSQ depression and HADS depression scales. Patients with panic disorder with agoraphobia had the highest scores on the 4DSQ anxiety scale, while patients with panic disorder without agoraphobia had the highest scores on the HADS anxiety scale. Furthermore, it is shown that patients with any anxiety disorder scored about the same as patients with major depression on both depression scales, but they scored higher than depressed patients on both anxiety scales. These observations can partly be attributed to overlap of the diagnostic categories (i.e. comorbidity). Of those patients with a depressive disorder diagnosis 45% had one or more comorbid anxiety disorder diagnoses. Of those patients with an anxiety disorder diagnosis 66% had a comorbid depressive disorder diagnosis.

**Table 2:** Distribution of CIDI/DSM-IV diagnoses, internal consistency of the scales (Cronbach's α), and mean scores (and standard deviations) of the scales per diagnostic category (n = 295)

		**questionnaire scales**				
		
**Diagnoses**	**number of patients (%)**	4DSQ distress (α 0.90)	4DSQ depression (α 0.86)	4DSQ anxiety (α 0.86)	HADS depression (α 0.83)	HADS anxiety (α 0.78)
Major depression	146 (49)	26.2 (4.9)	5.3 (3.3)	7.6 (5.5)	12.7 (3.4)	13.0 (3.7)

- mild	68 (23)	24.7 (5.2)	4.0 (2.7)	5.4 (4.7)	12.0 (3.3)	11.3 (3.4)

- moderate	36 (12)	26.4 (4.7)	5.3 (3.3)	8.7 (5.5)	12.5 (3.6)	13.7 (3.0)

- severe	42 (14)	28.4 (3.4)	7.5 (3.2)	10.2 (5.4)	14.1 (3.1)	15.0 (3.4)

Anxiety disorder	99 (34)	26.6 (5.2)	5.6 (3.4)	9.5 (5.5)	12.5 (3.4)	14.0 (3.2)

- generalized anxiety disorder	61 (21)	26.7 (4.9)	5.7 (3.3)	8.3 (5.4)	12.3 (3.0)	13.9 (3.1)

- panic disorder w/o agoraphobia	12 (4)	27.5 (4.5)	6.8 (3.8)	10.3 (3.5)	12.0 (3.8)	15.7 (2.7)

- panic disorder w/ agoraphobia	11 (4)	24.7 (5.8)	5.5 (3.4)	13.8 (4.7)	12.8 (3.9)	14.2 (3.9)

- agoraphobia w/o panic disorder	10 (3)	25.4 (8.2)	4.1 (3.2)	11.4 (7.6)	13.8 (4.6)	13.8 (3.9)

- social phobia	32 (11)	28.4 (3.6)	6.1 (3.1)	12.4 (5.5)	13.4 (3.8)	14.8 (3.0)

No depressive or anxiety disorder	115 (39)	18.3 (7.4)	2.2 (2.8)	3.3 (4.0)	8.1 (4.2)	9.4 (3.7)

4DSQ = Four-Dimensional Symptom Questionnaire

HADS = Hospital Anxiety and Depression Scale

w/= with, w/o = without

### ROC analyses

Table 3 presents the results of the ROC analyses. For four of the five comparisons there was no statistically significant difference between the 4DSQ and HADS scales. Only with respect to the ability to detect panic disorder, agoraphobia and social phobia, the 4DSQ anxiety scale performed significantly better than the HADS anxiety scale.

**Table 3:** Results of the Receiver Operating Characteristic (ROC) analyses of the 4DSQ and HADS scales with respect to detecting CIDI/DSM-IV depressive and anxiety disorders

**Diagnostic categories/levels**	**4DSQ scale**	**HADS scale**	**Z/p**
Any depressive/anxiety disorder	distress	total score	

	AUC 0.792 se 0.027	AUC 0.815 se 0.027	Z 0.525 p 0.596

Major depressive disorder	depression	depression	

- any major depressive disorder	AUC 0.745 se 0.029	AUC 0.762 se 0.028	Z 0.574 p 0.569

- moderate-severe major depressive disorder	AUC 0.780 se 0.029	AUC 0.739 se 0.030	Z 1.389 p 0.165

Anxiety disorder	anxiety	anxiety	

- any anxiety disorder	AUC 0.792 se 0.028	AUC 0.757 se 0.028	Z 1.333 p 0.184

- panic disorder/agoraphobia/social phobia	AUC 0.852 se 0.024	AUC 0.757 se 0.032	Z 3.294 **p 0.001**

4DSQ = Four-Dimensional Symptom Questionnaire

HADS = Hospital Anxiety and Depression Scale

AUC = area under the ROC curve

se = standard error

Z = standard Normal deviate

### Sensitivity, specificity, PPV and NPV

Table 4 shows the diagnostic performance of the 4DSQ distress score and the HADS total score with respect to the detection of any depressive or anxiety disorder. The 'optimal' cut-off point of ≥ 24 on the 4DSQ distress scale, where there is a balance between sensitivity and specificity, is found in the upper third of the scale. This indicates that severe distress is a hallmark of any CIDI/DSM-IV depressive or anxiety disorder. A lower cut-off point of ≥20 with a relatively high sensitivity of 0.90 can be used to exclude any depressive or anxiety disorder. The optimal cut-off point on the HADS total scale was ≥22 with a sensitivity of 0.76 and a specificity of 0.75. A lower cut-off point of ≥20 with a sensitivity of 0.86 can be used to exclude disorders (NPV 0.74), while a higher cut-off point of ≥25 with a specificity of 0.85 can be used to include disorders (PPV 0.85).

**Table 4:** Diagnostic performance of the 4DSQ distress scale and the HADS total score with respect to detecting any depressive or anxiety disorder (prevalence 61%)*

*4DSQ distress*	sensitivity	specificity	PPV	NPV
≥16	0.95	0.37	0.70	0.83

≥17	0.94	0.43	0.72	0.82

≥18	0.93	0.47	0.73	0.81

≥19	0.91	0.55	0.76	0.79

**≥20**	**0.90**	0.56	0.76	**0.78**

≥21	0.84	0.57	0.76	0.70

≥22	0.77	0.67	0.78	0.65

≥23	0.75	0.67	0.78	0.63

**≥24**	**0.71**	**0.73**	0.80	0.61

≥25	0.65	0.77	0.82	0.59

≥26	0.61	0.80	0.83	0.57

≥27	0.56	0.84	0.85	0.55

**≥28**	0.48	**0.85**	**0.83**	0.51

≥29	0.36	0.90	0.85	0.48

≥30	0.28	0.94	0.88	0.46

≥31	0.19	0.97	0.90	0.43

*HADS total score*				

≥16	0.96	0.41	0.72	0.86

≥17	0.93	0.46	0.73	0.80

≥18	0.92	0.50	0.74	0.80

≥19	0.89	0.59	0.77	0.78

**≥20**	**0.86**	0.64	0.79	**0.74**

≥21	0.82	0.68	0.80	0.71

**≥22**	**0.76**	**0.75**	0.82	0.66

≥23	0.71	0.80	0.85	0.64

≥24	0.63	0.84	0.86	0.59

**≥25**	0.56	**0.85**	**0.85**	0.55

≥26	0.46	0.88	0.85	0.51

≥27	0.39	0.89	0.84	0.48

≥28	0.32	0.91	0.85	0.46

≥29	0.27	0.92	0.84	0.45

≥30	0.21	0.95	0.86	0.43

* For each scale three cut-off points are highlighted in bold type: the optimal cut-off point with the best balance between sensitivity and specificity, the best lower cut-off point with a sensitivity ≥0.85, and the best higher cut-off point with a specificity ≥0.85

4DSQ = Four-Dimensional Symptom Questionnaire

HADS = Hospital Anxiety and Depression Scale

PPV = positive predictive value

NPV = negative predictive value

The diagnostic performance of the 4DSQ and HADS depression scales are shown in Table 5 for any major depressive disorder and Table 6 for moderate or severe major depressive disorder. The optimal 4DSQ depression cut-off point for any major depressive disorder was ≥4 while for moderate or severe major depressive disorder this cut-off point was ≥5. Lower cut-off points with sensitivity values of at least 0.85 to exclude caseness were ≥2 for any and ≥3 for moderate or severe major depressive disorder. A higher cut-off point with a specificity value of at least 0.85 to include caseness was ≥7 for both severity levels. The optimal HADS depression cut-off point for any major depressive disorder was ≥11 while for moderate to severe major depressive disorder this cut-off point was ≥12. Lower cut-off points with sensitivity values of at least 0.85 to exclude caseness were ≥9 for any and ≥10 for moderate or severe major depressive disorder. Higher cut-off points with specificity values of at least 0.85 to include caseness were ≥14 and ≥15.

**Table 5:** Diagnostic performance of the 4DSQ and HADS depression scales with respect to detecting any major depressive disorder (prevalence 49%)*

*4DSQ depression*	sensitivity	specificity	PPV	NPV
≥1	0.96	0.34	0.59	0.89

**≥2**	**0.88**	0.46	0.62	**0.80**

≥3	0.78	0.60	0.66	0.74

**≥4**	**0.65**	**0.72**	0.69	0.68

≥5	0.53	0.79	0.71	0.63

≥6	0.45	0.83	0.71	0.60

**≥7**	0.32	**0.87**	**0.71**	0.57

≥8	0.25	0.92	0.75	0.56

≥9	0.18	0.93	0.72	0.54

≥10	0.12	0.97	0.78	0.53

*HADS depression*				

≥7	0.97	0.30	0.57	0.92

≥8	0.93	0.39	0.60	0.84

**≥9**	**0.88**	0.49	0.63	**0.81**

≥10	0.84	0.60	0.68	0.80

≥**11**	**0.75**	**0.67**	0.69	0.73

≥12	0.60	0.75	0.70	0.65

≥13	0.49	0.78	0.69	0.61

≥**14**	0.40	**0.88**	**0.77**	0.60

≥15	0.32	0.89	0.73	0.57

≥16	0.21	0.93	0.74	0.55

≥17	0.16	0.96	0.79	0.54

* For each scale three cut-off points are highlighted in bold type: the optimal cut-off point with the best balance between sensitivity and specificity, the best lower cut-off point with a sensitivity ≥0.85, and the best higher cut-off point with a specificity ≥0.85

4DSQ = Four-Dimensional Symptom Questionnaire

HADS = Hospital Anxiety and Depression Scale

PPV = positive predictive value

NPV = negative predictive value

**Table 6:** Diagnostic performance of the 4DSQ and HADS depression scales with respect to detecting moderate and severe major depressive disorder (prevalence 26%)*

*4DSQ depression*	sensitivity	specificity	PPV	NPV
≥2	0.96	0.38	0.36	0.97

**≥3**	**0.87**	0.52	0.39	**0.92**

≥4	0.78	0.65	0.45	0.89

**≥5**	**0.64**	**0.73**	0.46	0.85

≥6	0.59	0.79	0.51	0.84

**≥7**	0.46	**0.87**	**0.55**	0.82

≥8	0.39	0.91	0.61	0.81

≥9	0.27	0.93	0.58	0.78

≥10	0.21	0.97	0.70	0.77

*HADS depression*				

≥8	0.96	0.30	0.33	0.96

≥9	0.91	0.38	0.35	0.92

**≥10**	**0.87**	0.48	0.37	**0.91**

≥11	0.81	0.56	0.40	0.89

**≥12**	**0.64**	**0.65**	0.40	0.84

≥13	0.56	0.72	0.42	0.82

≥14	0.49	0.82	0.49	0.82

**≥15**	0.39	**0.85**	**0.48**	0.79

≥16	0.27	0.90	0.50	0.77

≥17	0.22	0.95	0.59	0.77

* For each scale three cut-off points are highlighted in bold type: the optimal cut-off point with the best balance between sensitivity and specificity, the best lower cut-off point with a sensitivity ≥0.85, and the best higher cut-off point with a specificity ≥0.85

4DSQ = Four-Dimensional Symptom Questionnaire

HADS = Hospital Anxiety and Depression Scale

PPV = positive predictive value

NPV = negative predictive value

The diagnostic performance of the 4DSQ and HADS anxiety scales are shown in Table 7 for any anxiety disorder and Table 8 for panic disorder, agoraphobia and social phobia. The optimal 4DSQ anxiety cut-off point for any anxiety disorder was ≥6 while for panic disorder, agoraphobia and social phobia the optimal cut-off point was ≥8. Lower cut-off points with sensitivity values of at least 0.85 to exclude caseness were ≥4 for any anxiety disorder and ≥6 for panic disorder, agoraphobia and social phobia. Higher cut-off points with specificity values of at least 0.85 to include caseness were ≥9 and ≥10. The optimal HADS anxiety cut-off point for any anxiety disorder as well as for panic disorder, agoraphobia and social phobia was ≥13. Lower cut-off points with sensitivity values of at least 0.85 to exclude caseness were ≥10 for any anxiety disorder and ≥11 for panic disorder, agoraphobia and social phobia. A higher cut-off point with a specificity value of at least 0.85 to include caseness was ≥16 for both severity levels of anxiety disorders.

**Table 7:** Diagnostic performance of the 4DSQ and HADS anxiety scales with respect to detecting any anxiety disorder (prevalence 34%)*

*4DSQ anxiety*	sensitivity	specificity	PPV	NPV
≥1	0.98	0.20	0.38	0.95

≥2	0.94	0.33	0.41	0.91

≥3	0.90	0.45	0.45	0.90

**≥4**	**0.85**	0.57	0.50	**0.88**

≥5	0.75	0.64	0.51	0.83

**≥6**	**0.70**	**0.72**	0.56	0.82

≥7	0.67	0.78	0.60	0.82

≥8	0.65	0.84	0.67	0.82

**≥9**	0.59	**0.88**	**0.71**	0.81

≥10	0.54	0.90	0.73	0.79

≥11	0.40	0.91	0.70	0.75

≥12	0.32	0.93	0.71	0.73

≥13	0.24	0.94	0.67	0.71

≥14	0.20	0.95	0.67	0.70

*HADS anxiety*				

≥8	0.98	0.27	0.40	0.96

≥9	0.98	0.35	0.43	0.97

**≥10**	**0.91**	0.43	0.45	**0.90**

≥11	0.84	0.51	0.46	0.86

≥12	0.78	0.63	0.51	0.85

**≥13**	**0.65**	**0.70**	0.52	0.80

≥14	0.58	0.78	0.57	0.78

≥15	0.47	0.83	0.58	0.75

**≥16**	0.32	**0.89**	**0.59**	0.72

≥17	0.21	0.93	0.62	0.70

≥18	0.15	0.97	0.71	0.69

* For each scale three cut-off points are highlighted in bold type: the optimal cut-off point with the best balance between sensitivity and specificity, the best lower cut-off point with a sensitivity ≥0.85, and the best higher cut-off point with a specificity ≥0.85

4DSQ = Four-Dimensional Symptom Questionnaire

HADS = Hospital Anxiety and Depression Scale

PPV = positive predictive value

NPV = negative predictive value

**Table 8:** Diagnostic performance of the 4DSQ and HADS anxiety scales with respect to detecting panic disorder, agoraphobia and social phobia (prevalence 19%)*

*4DSQ anxiety*	sensitivity	specificity	PPV	NPV
≥4	0.98	0.53	0.33	0.99

≥5	0.89	0.61	0.35	0.96

**≥6**	**0.86**	0.68	0.39	**0.95**

≥7	0.82	0.73	0.42	0.95

**≥8**	**0.79**	**0.78**	0.46	0.94

≥9	0.71	0.82	0.49	0.92

**≥10**	0.64	**0.85**	**0.49**	0.91

≥11	0.54	0.89	0.53	0.89

≥12	0.43	0.91	0.53	0.87

≥13	0.32	0.93	0.50	0.85

≥14	0.27	0.94	0.50	0.85

≥15	0.23	0.96	0.56	0.84

*HADS anxiety*				

≥8	1.00	0.23	0.23	1.00

≥9	1.00	0.29	0.25	1.00

≥10	0.95	0.38	0.26	0.97

**≥11**	**0.86**	0.45	0.27	**0.93**

≥12	0.80	0.56	0.30	0.92

**≥13**	**0.68**	**0.65**	0.31	0.90

≥14	0.63	0.73	0.35	0.89

≥15	0.57	0.80	0.40	0.89

**≥16**	0.41	**0.87**	**0.43**	0.86

≥17	0.27	0.92	0.44	0.84

≥18	0.20	0.96	0.52	0.84

* For each scale three cut-off points are highlighted in bold type: the optimal cut-off point with the best balance between sensitivity and specificity, the best lower cut-off point with a sensitivity ≥0.85, and the best higher cut-off point with a specificity ≥0.85

4DSQ = Four-Dimensional Symptom Questionnaire

HADS = Hospital Anxiety and Depression Scale

PPV = positive predictive value

NPV = negative predictive value

## Discussion

### Main findings

We examined and compared the abilities of the 4DSQ and the HADS to detect CIDI/DSM-IV defined depressive and anxiety disorders in distressed patients in primary care. Regarding any major depressive or any anxiety disorder, we found that the 4DSQ and the HADS depression and anxiety scales yielded comparable and satisfactory results with AUC values well above 0.70. Moreover, the 4DSQ distress scale turned out to be as effective as the HADS total score in detecting any depressive or anxiety disorder, which is in agreement with the conceptualization of distress as the most basic expression of human psychological suffering. However, in distressed patients, it is specifically important to identify the more severe disorders as these patients may be in need for specific treatment. Therefore, we looked at how well the 4DSQ and the HADS detected moderate and severe major depressive disorder and panic disorder, agoraphobia and social phobia. As it turned out, the 4DSQ anxiety scale performed significantly better than the HADS anxiety scale while the 4DSQ depression scale performed a little bit better than the HADS depression scale but the difference was statistically not significant. Given the content difference between the 4DSQ and HADS scales, and the assumption that the 4DSQ items refer to more severe symptoms than the HADS items, these findings are not particularly surprising.

Furthermore, the cut-off points that we established for the 4DSQ depression scale compared reasonably well with the recommended cut-off points of ≥3 en ≥6. However, the cut-off points that we established for the 4DSQ anxiety scale appeared to be substantially lower than the recommended cut-off points of ≥8 en ≥13, and, therefore, the cut-off points of the 4DSQ anxiety scale may need downward revision. In contrast, the cut-off points that we established for the HADS depression and anxiety scales proved to be substantially higher than the recommended cut-off points of both HADS scales. Therefore, the HADS cut-off points may need upward revision, perhaps to ≥9 and ≥14 for depression, and ≥10 and ≥16 for anxiety.

### Comparison with other studies

The present 4DSQ results can be compared with the only previous 4DSQ validation study published [18] in which the performance of the depression scale was studied in distressed primary care patients. This yielded an AUC value of 0.83 and an optimal cut-off point of ≥6. In the present study we found slightly lower AUC values and lower optimal cut-off points, which is probably due to a more severe case mix and a different reference standard, the Short Depression Interview [33] in the former study. The performance of the anxiety scale was studied in anxious primary care patients yielding an AUC value of 0.66 and an optimal cut-off point of ≥10. In the present study we found significantly higher AUC values and lower optimal cut-off points, which is very likely related to an extremely severe case mix (87% of the patients had one or more DSM-IV anxiety disorders), little contrast between the groups, and a different reference standard, the Structured Clinical Interview for DSM-IV (SCID) [34] in the former study.

Our results regarding the HADS can be compared with four primary care studies [35-38]. All studies used samples of consecutive (i.e. unselected) patients. The study of Lam et al. [35] was conducted in older patients (≥60 years). Olssøn et al. [37] used self-report measures as reference standards; the other studies used (standardized) psychiatric interviews. Optimal cut-off points for the HADS depression scale were reported to vary between ≥4 and ≥7, and for the HADS anxiety scale between ≥3 and ≥9. In the present study we found significantly higher optimal cut-off points: ≥11 for any depressive disorder and ≥ 13 for any anxiety disorder. These differences may be explained by differences in study populations (case mix) [39,40], reference standards and language versions of the HADS. A study population, like consecutive primary care patients, that includes a large proportion of healthy individuals, causes the optimal cut-off point to shift downwards. However, screening unselected people is far less efficient than screening high risk groups such as patients presenting with psychological symptoms or reasons for encounter.

### Limitations and strengths

Our study sample was limited by the selection of distressed general practice patients (considered to have a 'nervous breakdown'), aged 18–60 years, being employed, and being on sick leave. Therefore, some caution should be observed in generalizing our results to distressed older patients (>60 years), distressed patients without paid employment, and employed distressed patients who are not on sick leave. Moreover, even more caution should be observed in generalizing our results to patients who do not disclose their distress to their GP. Such patients, who nevertheless have a relatively high risk for depressive and anxiety disorders, are, in particular, patients with medically unexplained physical symptoms [41] and patients with chronic somatic diseases [42]. Future research should clarify whether the results of the present study can be generalized to these important patient groups.

The GPs were instructed to exclude patients with obvious (i.e. already diagnosed) depressive or anxiety disorders. Recognizing that case mix differences may impact the performance characteristics of diagnostic tests [39], let us consider how our results could have looked like if we had not excluded these patients. Patients with GP diagnosed depressive and anxiety disorders would probably have had more severe symptoms and disorders than those included in the present study sample. Therefore, they would have had a relatively high probability of fulfilling the DSM-IV criteria for caseness (according to the CIDI interview), and a smaller chance of obtaining false-negative test results than the present sub-sample of CIDI-DSM-IV cases in the study sample. Addition of such a more severe group of patients to our sample would, therefore, have resulted in increased sensitivity values as relatively more cases would have been correctly classified. In addition, because of the rise in prevalence, the positive predictive values would have increased and the negative predictive values would have decreased. Moreover, due to an increase in contrast between cases and non-cases, the diagnostic accuracy (AUC values) would probably have increased, and due to a shift in the severity spectrum, the optimal cut-off points would probably have shifted to higher values. In other words, it is unlikely that the exclusion of patients with obvious depressive and anxiety disorders in our study has resulted in an overestimation of the diagnostic accuracy of the questionnaires.

The exclusion of patients with obvious depressive and anxiety disorders can also be considered to be a strength of the study. Considering that a diagnostic instrument is meant to be used in situations where there is a diagnostic problem, diagnostic research should be performed in relevant patient populations (the 'relevant domain') [40]. With respect to detecting depressive and anxiety disorders, there is no diagnostic problem in patients in whom depressive and anxiety disorders are clearly discernable. When the patients' depressive and anxiety disorders are obvious, the application of tools to detect these disorders is superfluous. In the present study, we have selected a relevant population of distressed patient, in whom depressive and anxiety disorders often remain undetected as such [6].

### Further considerations

In our study, GPs were requested to recruit 'nervous breakdown' patients on sick leave, and to exclude patients with readily diagnosable depressive and anxiety disorders. Nevertheless, when the patients were subsequently subjected to a standardized diagnostic interview (the CIDI), one third of them (34%) received a diagnosis of moderate or severe major depressive disorder and/or panic disorder, agoraphobia or social phobia. As much as 61% of the patients received any DSM-IV depressive or anxiety disorder diagnosis. Our results thus illustrate that, when patients present themselves with a story of a 'nervous breakdown', GPs tend to overlook depressive and anxiety disorders, even those that are severe enough to justify a specific diagnosis and treatment. Our concerns explicitly regard the latter group, the 26% of the patients with a moderate or severe major depressive disorder and the 19% of the patients with a panic disorder, agoraphobia or social phobia (together 34% of the sample). We do not worry about the patients with mild major depressive disorders as they probably would not benefit from a specific antidepressant treatment [43,44]. We do recognize that these 'mild' patients need their distress to be recognized and managed, but they do not need to be given a label of 'major depressive disorder'. We do not worry either about patients with 'generalized anxiety disorder' as the essence of this diagnosis is distress for a period of more than six months. In a sample of distressed patients not all patients with distress for more than six months are chronic worriers; some or perhaps most of them are just confronted with long-lasting social difficulties and stressors, and, although we recognize that these patients need their distress to be recognized and taken care of, most of them are unlikely to benefit from a diagnosis of 'anxiety disorder'.

In addition, there is one more argument in favour of refraining from screening for just any depressive or anxiety disorder (as opposed to more severe disorders only) in a sample of relatively severely distressed patients (who have gone on sick leave) in primary care. The argument is that, in this particular case, screening is not very successful in separating out patients with probabilities for caseness small enough to justify no further investigation or follow-up. We have seen that, in this sample with a prevalence of 61% for any depressive or anxiety disorder (49% for any major depressive disorder and 34% for any anxiety disorder), it is difficult to exclude caseness because of relatively low predictive values of negative scores (NPV; see Table 4). If, for some reason, one insists on diagnosing any DSM-IV depressive or anxiety disorder, one virtually has to investigate every patient in a situation where the prior probability of being a case is as high as 61%. On the other hand, if one is dealing with a sample comprising more mildly, and perhaps less obviously distressed patients, one might be interested in detecting any DSM-IV depressive or anxiety disorder in order to find patients distressed severely enough to warrant special attention (not specific treatment), then the HADS (both the depression and anxiety scales, or the total scale) can be used for that purpose, as well as the 4DSQ distress scale. The distress scale measures 'normal' everyday distress in the score range 0–10. From the score of 11 upwards the distress scale measures moderate to severe distress that is associated with (occupational) stress, social dysfunctioning and sickness absence [45,46]. Our findings suggest that in the score range 11–20 there is distress with a relatively small probability of having any DSM-IV defined depressive or anxiety disorder, while in the range 21–32 there is an increasing probability for such a disorder.

## Conclusion

In a sample of distressed general practice patients, the 4DSQ and the HADS are both able to detect any DSM-IV depressive or anxiety disorder. However, when one is specifically interested in detecting cases severe enough to warrant specific treatment (e.g. antidepressant pharmacotherapy or specialized cognitive behavioural therapy), the 4DSQ has some advantage over the HADS, especially for the detection of panic disorder, agoraphobia and social phobia. Future research should focus on the diagnostic accuracy in high-risk groups, in particular in patients presenting their distress to their GP, patients with medically unexplained physical symptoms and patients with chronic physical conditions. Special attention needs to be given to optimal cut-off points in these groups as these may very well differ from those determined in unselected primary care patients.

## Competing interests

BT is the copyright owner of the 4DSQ and receives copyright fees from companies that use the 4DSQ on a commercial basis (the 4DSQ is freely available for non-commercial use in health care and research). BT received fees from various institutions for workshops on the application of the 4DSQ in primary care settings.

## Authors' contributions

BT participated in the study design, performed the statistical analyses and drafted the manuscript. EPMB acquired the data and assisted in the analyses. PFMV conceived of the study, and participated in its design and coordination. BT, HWJM, and HEH conceived the idea for the manuscript. EPMB and HWJM helped to draft the manuscript. PFMV and HEH critically revised the manuscript. All authors read and approved the final manuscript.

## Pre-publication history

The pre-publication history for this paper can be accessed here:


